# Quantitative proteomic analysis of cultured skin fibroblast cells derived from patients with triglyceride deposit cardiomyovasculopathy

**DOI:** 10.1186/1750-1172-8-197

**Published:** 2013-12-21

**Authors:** Yasuhiro Hara, Naoko Kawasaki, Ken-ichi Hirano, Yuuki Hashimoto, Jun Adachi, Shio Watanabe, Takeshi Tomonaga

**Affiliations:** 1 Laboratory of Proteome Research, National Institute of Biomedical Innovation 7-6-8 Saito-Asagi, Ibaraki City, Osaka 567-0085, Japan; 2 Department of Cardiovascular Medicine, Graduate School of Medicine, Osaka University, 2-2 Yamadaoka, Suita, Osaka 565-0871, Japan

**Keywords:** Proteome, Triglyceride deposit cardiomyovasculopathy, SILAC, SRM/MRM, ATGL, Rare disease

## Abstract

**Background:**

Triglyceride deposit cardiomyovasculopathy (TGCV) is a rare disease, characterized by the massive accumulation of triglyceride (TG) in multiple tissues, especially skeletal muscle, heart muscle and the coronary artery. TGCV is caused by mutation of adipose triglyceride lipase, which is an essential molecule for the hydrolysis of TG. TGCV is at high risk for skeletal myopathy and heart dysfunction, and therefore premature death. Development of therapeutic methods for TGCV is highly desirable. This study aims to discover specific molecules responsible for TGCV pathogenesis.

**Methods:**

To identify differentially expressed proteins in TGCV patient cells, the stable isotope labeling with amino acids in cell culture (SILAC) method coupled with LC-MS/MS was performed using skin fibroblast cells derived from two TGCV patients and three healthy volunteers. Altered protein expression in TGCV cells was confirmed using the selected reaction monitoring (SRM) method. Microarray-based transcriptome analysis was simultaneously performed to identify changes in gene expression in TGCV cells.

**Results:**

Using SILAC proteomics, 4033 proteins were quantified, 53 of which showed significantly altered expression in both TGCV patient cells. Twenty altered proteins were chosen and confirmed using SRM. SRM analysis successfully quantified 14 proteins, 13 of which showed the same trend as SILAC proteomics. The altered protein expression data set was used in Ingenuity Pathway Analysis (IPA), and significant networks were identified. Several of these proteins have been previously implicated in lipid metabolism, while others represent new therapeutic targets or markers for TGCV. Microarray analysis quantified 20743 transcripts, and 252 genes showed significantly altered expression in both TGCV patient cells. Ten altered genes were chosen, 9 of which were successfully confirmed using quantitative RT-PCR. Biological networks of altered genes were analyzed using an IPA search.

**Conclusions:**

We performed the SILAC- and SRM-based identification-through-confirmation study using skin fibroblast cells derived from TGCV patients, and first identified altered proteins specific for TGCV. Microarray analysis also identified changes in gene expression. The functional networks of the altered proteins and genes are discussed. Our findings will be exploited to elucidate the pathogenesis of TGCV and discover clinically relevant molecules for TGCV in the near future.

## Background

Adipose triglyceride lipase (ATGL) deficiency causes the onset of neutral lipid storage disease with myopathy (NLSDM), a rare genetic disorder which is transmitted as an autosomal recessive trait [1,2]. Patients with NLSDM have either homozygous or compound heterozygous mutations in the ATGL gene. ATGL catalyzes the rate-limiting step in the hydrolysis of TG stored in lipid droplets [3,4]. ATGL deficiency is characterized by the presence of intracellular triglyceride (TG) deposition in most tissues, including leukocytes (Jordans’ anomaly), skeletal muscles and the heart [1,2]. Clinically, the patients reported so far show primarily skeletal muscle weakness associated with myopathy, with highly elevated creatine kinase levels, and show cardiomyopathy usually observed at later stages of the disease [2,5].

One of the NLSDM phenotypes, discovered in Japan, shows massive TG accumulation in both the coronary artery and myocardium, resulting in severe heart failure. This symptom has been designated “Triglyceride deposit cardiomyovasculopathy (TGCV)” [6,7]. Cardiomyopathy is lethal in patients with TGCV and necessitates cardiac transplantation; thus, therapeutic methods for reducing the burden on patients with this intractable disease are highly desirable. In addition, sensitive noninvasive biomarkers are needed for monitoring the therapeutic response rather than diagnostic biomarkers for detecting this disease. A clear understanding of the pathogenesis of TGCV is essential to exploit the therapeutic methods. However, the molecular nature of TGCV remains unknown to date.

As proteins are almost always the effectors of cellular functions, the use of proteomic approaches to decipher the molecular basis of diseases might offer new insight. Therefore, in this study, we performed proteomic analysis to examine differentially expressed proteins in TGCV patient cells. We employed the stable isotope labeling with amino acids in cell culture (SILAC) method coupled with LC-MS/MS [8] and performed large-scale quantitative proteomic analysis.

The list of differentially expressed proteins obtained by comprehensive quantitative analysis needs to be validated, but this step is an expensive and time-consuming process requiring a pre-existing antibody. A more recent mass spectrometry-based technique called selected/multiple reaction monitoring (SRM/MRM) is a useful method for the validation of proteins without antibodies [9]. In this study, we confirmed differentially expressed proteins identified by SILAC proteomics using SRM/MRM. As a result, we identified for the first time differentially expressed proteins between TGCV patient and healthy volunteer cells. Our identified proteins will be useful for elucidation of the pathogenesis of TGCV and the exploitation of therapeutic methods for TGCV in the future.

## Methods

### Cell culture

Skin fibroblast cells were isolated from the skin of two TGCV patients in the Department of Cardiovascular Medicine, Osaka University Graduate School of Medicine, Japan. The local ethics committee approved the study and informed consent was obtained from the donors. Cells were cultured in Iscove’s modified Dulbecco’s medium (IMDM) supplemented with 10% fetal bovine serum (FBS; Invitrogen, Carlsbad, CA, USA) and penicillin/streptomycin (100 IU/ml). For SILAC experiments, Dulbecco's modified Eagle’s medium without L-arginie and L-lysine (Invitrogen) was supplemented with dialyzed FBS (Invitrogen). The medium was then divided into three portions and supplemented with ^13^C_6,_^15^ N_4_ L-arginine and ^13^C_6_, ^15^ N_2_ L-lysine or ^13^C_6_ L-arginine and ^4,4,5,5^D_4_ L-lysine or normal L-arginine and L-lysine, to produce “heavy” or “medium” or “light” SILAC medium, respectively. All isotopes labeled L-arginine and L-lysine were purchased from Cambridge Isotope Laboratories (Tewksbury, MA, USA). Skin fibroblast cells were grown in SILAC medium for at least 6 doubling times to ensure that the amino acids had been fully incorporated.

### Oil red O lipid staining

Cells were cultured on chamber slides (BD Biosciences, San Jose, CA, USA). The staining solution was prepared by dissolving 0.5 g Oil Red O powder (Sigma-Aldrich, St Louis, MO, USA) in 100 ml isopropanol, followed by 1:1 dilution with distilled water. Then, the solution was allowed to stand for 10 min before it was filtered through filter paper. The cells were washed in PBS, fixed with 10% formalin at room temperature for 10 min, and stained with Oil Red O at room temperature for 20 min. Samples were washed with distilled water and counterstained with Mayer's hematoxylin (Wako Pure Chemical Industries, Osaka, Japan) for 2 min.

### Preparation of protein samples, 1-D SDS-PAGE separation and in-gel trypsin digestion

Cells were scraped into ice-cold RIPA buffer containing 50 mM Tris–HCl, pH 8.0, 150 mM NaCl, 1% Nonidet P-40, 0.5% sodium deoxycholate, 0.1% SDS, with 1 × protease inhibitor, compete Mini (Roche Applied Science, Indianapolis, IN, USA) and agitated at 4°C for 30 min. After centrifugation at 14000 rpm at 4°C for 20 min, lysates were collected and protein concentrations were determined using a DC Protein Assay (Bio-Rad, Hercules, CA, USA). Equal amounts (in weight) of lysates from three light (Arg0, Lys0) labeled control cells were pooled to generate the control, and then lysates of three labeled types, light (Arg0, Lys0), medium (Arg6, Lys4) and heavy (Arg10, Lys8), were mixed in equal amount (in weight). A 100 μg sample of mixed proteins was separated by SDS-PAGE using NuPAGE Novex 4-12% Bis-Tris (Invitrogen). Bands were visualized with Gels Simply Blue Safe Stain (Invitrogen) and lanes were sliced into 46 sections. After distaining with 50% ethanol in 50 mM ammonium bicarbonate (ABC), proteins in gel pieces were reduced with 10 mM DTT in ABC and alkylated by 50 mM iodoacetamide in ABC. After gel dehydration with 100% ethanol, the gel pieces were covered with approximately ~40 μl of 12.5 μg/mL trypsin in ABC and in-gel digestion was performed at 37°C for 16 h. Peptides were extracted from gels with 3% trifluoroacetic acid (TFA), 30% acetonitrile (ACN) and then 100% ACN. The resulting peptide mixtures were dried and resolved with 0.1% TFA and 2% ACN, and desalted using C18 stage Tips [10].

### NanoLC-MS/MS

NanoLC-MS/MS analysis was conducted using an LTQ-Orbitrap Velos mass spectrometer (Thermo Fisher Scientific, Bremen, Germany) equipped with a nanoLC interface (AMR, Tokyo, Japan), a nano HPLC system (Paradigm MS2; Michrom Bioresources, Auburn, CA, USA), and an HTC-PAL autosampler (CTC Analytics, Zwingen, Switzerland). L-column2 C18 particles (Chemicals Evaluation and Research Institute, Kurume, Japan) were packed into a self-pulled needle (200 mm length × 100 μm inner diameter) using a Nanobaume capillary column packer (Western Fluids Engineering, Wildomar, CA, USA). The mobile phases consisted of (A) 0.1% formic acid and 2% ACN and (B) 0.1% formic acid and 90% ACN. The peptides dissolved in 2% ACN and 0.1% TFA were loaded onto a trap column (0.3 × 5 mm, L-column ODS; CERI, Japan). The nanoLC gradient was delivered at 500 nl/min and consisted of a linear gradient of mobile phase B developed from 5 to 35% in 135 min. A spray voltage of 2000 V was applied.

### Data acquisition with LTQ-Orbitrap Velos

Full MS scans were performed in the orbitrap mass analyzer of LTQ-Orbitrap Velos (scan range 350–1500 m/z, with 30 K full width at half maximum (FWHM) resolution at 400 m/z). In MS scans, the 10 most intense precursor ions were selected for MS/MS scans with the LTQ-Orbitrap Velos. The dynamic exclusion option was implemented with a repeat count of 1 and exclusion duration of 60 s. This was followed by collision-induced dissociation (CID) MS/MS scans of the selected ions performed in the linear ion trap mass analyzer. The values of automated gain control (AGC) were set to 1.00 × 10^6^ for full MS and 1.00 × 10^4^ for CID MS/MS. Normalized collision energy values were set to 35%.

### Protein identification and quantification using MaxQuant software

The resulting mass spectra were analyzed using MaxQuant Software (version 1.2.2.5) [11]. In short, raw MS files were loaded directly into MaxQuant, and identification and quantitation of individual peptides were generated in protein groups. The MaxQuant searches were executed using the International Protein Index (IPI) human protein database (version 3.67, 87302 forward and 87302 reverse protein sequences). All entries were filtered using a false positive rate of 1%. The following search parameters were used: two missed cleavages permitted, carbamidomethylation on cysteine fixed modification, and oxidation (methionine) and acetyl (N-terminus proteins) variable modifications. The mass tolerances for precursor ions and fragment ions were 20 ppm and 0.5 Da, respectively. Quantification of proteins was based on the normalized heavy/light (H/L) and medium/light (M/L) ratios as determined by MaxQuant.

### Stable isotope-labeled peptides

Proteotypic peptides were chosen based on SILAC proteomics data. For SRM/MRM analysis of the 20 target proteins, 42 stable isotope-labeled peptides (SI-peptides, crude peptide: approximately 50% peptide purity and >99% isotope purity; Greiner Bio One, Frickenhausen, Germany) were synthesized. SI-peptides had isotope-labeled lysine or arginine at their C-terminus. Each SI-peptide was dissolved in distilled 40% ACN and 0.1% TFA, and stored at −80°C.

### Creation of SRM/MRM transition list using SI-peptides

The mixture of SI-peptides was analyzed by LC-MS/MS using LTQ-Orbitrap XL (CID mode), and an msf file was generated using Proteome Discoverer and Mascot. The msf file was opened with Pinpoint software (version 2.3.0; Thermo Fisher Scientific), and a list of MS/MS fragment ions derived from SI-peptides was generated. Four MS/MS fragment ions were selected for SRM/MRM transitions of each targeted peptide based on the following criteria: y-ion series, strong ion intensity and at least 2 amino acids in length.

### Optimization and operation of SRM/MRM method

SRM/MRM methods for each SI-peptide were created by Pinpoint 1.0, which included SRM/MRM transition lists and the instrument method with the following parameters: scan width of 0.002 m/z, Q1 resolution of 0.7 FWHM, cycle time of 1 s, and gas pressure of 1.8 mTorr. The SI-peptide mixture was analyzed by LC-SRM/MRM on the TSQ-Vantage triple quadruple mass spectrometer (Thermo Fisher Scientific) equipped with the parameters mentioned above. The nanoLC gradient was delivered at 500 nl/min and a spray voltage of 1900–2000 V was applied. Test runs of the SI-peptide mixture were performed to establish the retention time window (±2 min) for each peptide ion and optimize the collision energy for each transition. Four transitions were chosen for each peptide and all fragment ions were y-ions. When possible, two peptides were used per protein and all SRM/MRM analyses were run in duplicate. For SRM/MRM analyses, proteins were prepared as follows. Skin fibroblast cells cultured in normal IMDM medium were harvested, and proteolytic digestion were performed by a phase-transfer surfactant (PTS) protocol using an MPEX PTS reagent kit (GL Sciences, Tokyo, Japan) [12]. Briefly, pellets were lysed with PTS B buffer followed by sonication for 5 min using a Bioruptor sonicator (Cosmo Bio, Tokyo, Japan). Lysed proteins were reduced with 10 mM DTT, alkylated with 50 mM iodoacetamide and sequentially digested by 1:100 (w/w) trypsin for 16 h at 37°C. An equal volume of ethylacetate was added to the digested samples, and the mixtures were acidified by 1% TFA followed by vortexing to transfer the detergents to the organic phase. After centrifugation, the aqueous phase containing peptides was collected. The resulting peptide mixtures were desalted using C18 stage Tips and resolved with 0.1% TFA and 2% ACN. Two micrograms of digested sample were transferred to a new tube and the SI-peptide mixture was added. The amount of SI-peptide was optimized to achieve similar ion intensity to the corresponding endogenous peptide, if possible. Samples were analyzed by LC-SRM/MRM on the TSQ-Vantage using the optimized SRM/MRM method.

### SRM/MRM data analysis

SRM/MRM data were processed using Pinpoint 1.0. The peak area in the chromatogram of each SRM/MRM transition was calculated, and the values of endogenous targeted peptides were normalized to those of the corresponding SI-peptides. When the transition profile was different between endogenous peptides and SI-peptides, the transition was excluded from the quantification process. The different transition profile might have been caused by the detection of untargeted peptides. In addition, transitions having signal-to-noise ratios (S/N) of <10 were discarded from this study. In such cases, only peptides having more than one transition were used.

Protein accession numbers identified by SILAC proteomics analysis and their corresponding fold changes were imported into Ingenuity Pathway Analysis (IPA) software (Ingenuity Systems, Redwood City, CA, USA, http://www.ingenuity.com) for network and functional analysis. IPA is web-based software that constructs protein interaction networks in silico based upon published associations that have been collated from the literature.

### Microarray analysis

Skin fibroblast cells derived from two TGCV patient cells and two control cells (LC1, LC2, control1 and control2) were cultured in SILAC medium. Cells were harvested, and total RNA was isolated using a QIA shredder (Qiagen, Valencia, CA, USA) and an RNeasy Mini Kit (Qiagen) according to the manufacturer’s instructions. The quantity and purity of RNA were evaluated using a NanoDrop (Nanodrop Technologies, Wilmington, DE, USA) and an Agilent 2100 Bioanalyzer (Agilent Biotechnologies, Santa Clara, CA, USA). RNA from two control cells was equally mixed prior to array analysis. Analysis using Human Genome U133 Plus 2.0 microarray (Affymetrix, Santa Clara, CA, USA) was performed according to the standard Affymetrix protocol. Briefly, total RNA was reverse-transcribed into complementary DNA (cDNA) and cDNA was in vitro transcribed to biotinylated RNA. After fragmentation, 12.5 μg biotinylated RNA was hybridized overnight to the array, and then arrays were washed, stained with streptavidin–phycoerythrin and scanned using a GeneChip 3000 7G scanner (Affymetrix). The acquisition and initial quantification of array images were conducted using GeneChip Command Console Software (Affymetrix), then data were analyzed by GeneSpring GX Software (Affymetrix). We used present and absent call filtering to determine which transcripts were able to be considered accurately measured, which is a standard method for microarray analysis [13].

### Real-time reverse transcription PCR

Total RNA was isolated from skin fibroblast cells using the same method as described above, except that cells were cultured in normal medium. Complementary DNA was synthesized using the First Strand cDNA Synthesis Kit for RT-PCR (AMV) (Roche Applied Science). Real-time reverse transcription (RT) -PCR was performed using the Power SYBR Green PCR Master Mix (Applied Biosystems, Foster City, CA, USA) and ABI Prism 7900HT (Applied Biosystems). The following PCR primers were used: *FBN2*, 5′-GAAGTATTCATGAACCTGATC-3′ (forward) and 5′-GGTTGAACTTCATGTTGACGG-3′ (reverse); *CXCR7*, 5′-ATGTCACACAGTGCCTGTCGC-3′ (forward) and 5′-ATGAAGGCCTTCATCAGCTCG-3′ (reverse); *PLA2G4A*, 5′-ACCCAAGAATCCTGATATGGAG-3′ (forward) and 5′-CCTGGAGCCTTGTACTTTCTG-3′ (reverse); *FLG*, 5′-TAGACACTCTCAGCACGGAAGTG-3′ (forward) and 5′-CCTGGGTCCTTATTAATATACG-3′ (reverse); *FBL2*, 5′-GGGAACTGTGGGCTGTACTAC-3′ (forward) and 5′-AAATCCCATTACGGACACCTCT-3′ (reverse); *RAB27B*, 5′-CCTACCAGATCAGAGGGAAGTC-3′ (forward) and 5′-CATTCTGTCCAGTTGCTGCAC-3′ (reverse); *MC4R*, 5′-AGGTGCCAATATGAAGGGAGCG-3′ (forward) and 5′-GGATTCTGAGGACAAGAGATGTAG-3′ (reverse); *SLC16A6*, 5′-TCAGAGCATAGCAGGACTGGC-3′ (forward) and 5′-AGGCCCTGCTGTAGATCTTAC-3′ (reverse); *TNFRSF21*, 5′-TCTCCGCTGTGACTCTACATC-3′ (forward) and 5′-TACCTGCCGCAACACTGTGTC-3′ (reverse); *NCAM1*, 5′-GAAAGATGAGTCCAAGGAGCC-3′ (forward) and 5′-TCCGTCAGTGGCGTGGTCTCG-3′ (reverse); *PLIN2*, 5′-TTGGATATGATGATACTGATG-3′ (forward) and 5′-ACGTGGTCTGGAGCTGCTGAG-3′ (reverse); *RPS18*, 5′-TTTGCGAGTACTCAACACCAAC-3′ (forward) and 5′-AGCATATCTTCGGCCCACACC-3′ (reverse). The relative mRNA levels of each gene were normalized to *RPS18* expression.

## Results

### Identification of proteins differentially expressed in TGCV patient cells

**Figure 1:**
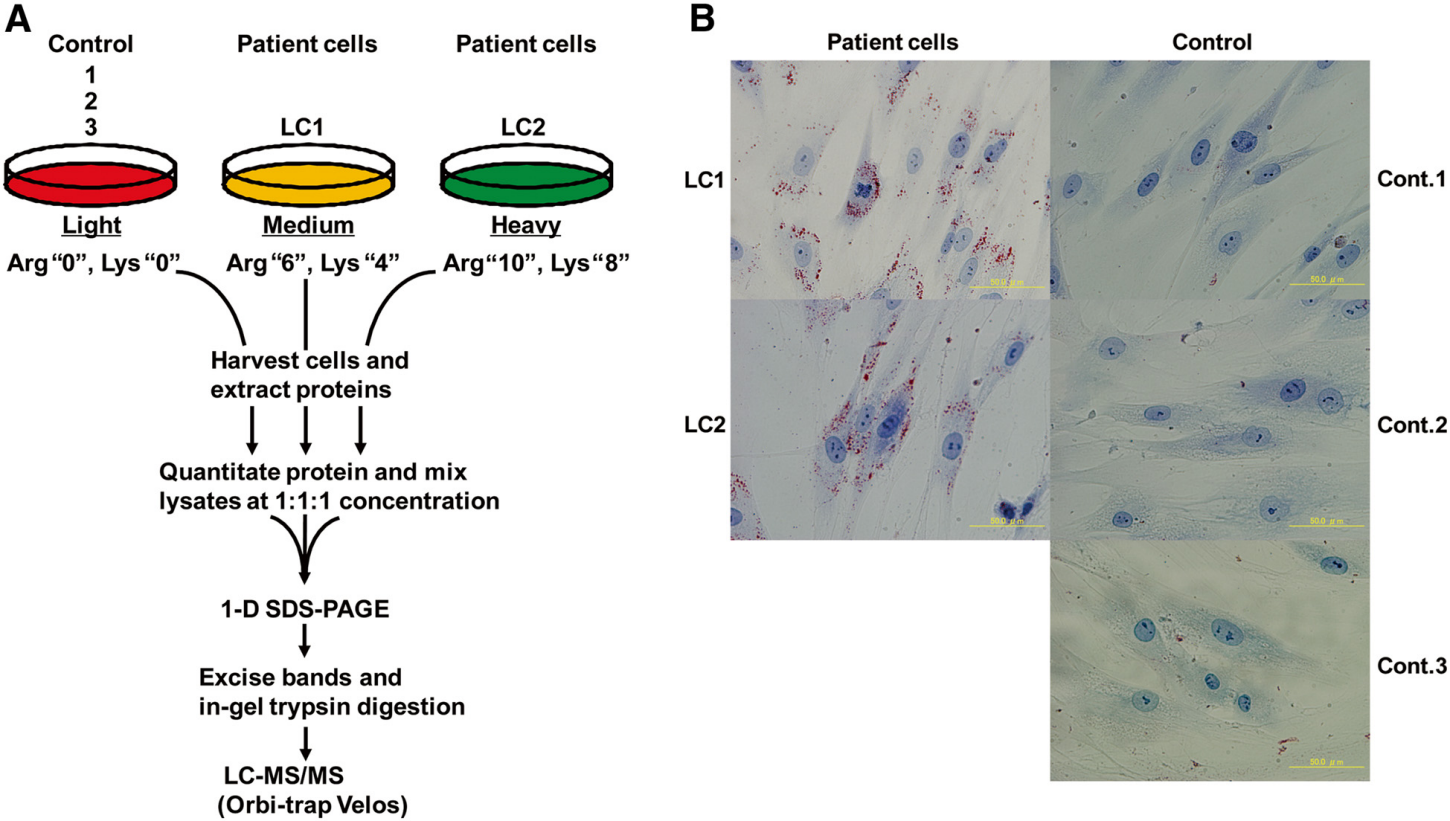
**SILAC-based quantitative proteomics. (A)** Flowchart of triple SILAC coupled with LC-MS/MS for the comparative analysis of three distinct cell populations. **(B)** Oil Red O staining of SILAC-labeled skin fibroblast cells derived from TGCV patients and healthy volunteers (controls). Characters on left and right sides of panels indicate names of patient and control cells, respectively.

We have applied a quantitative proteomics approach using skin fibroblast cells to identify proteins differentially expressed between TGCV patients and healthy volunteers (control). Tissue samples from primary lesions such as skeletal and cardiac muscles would have been ideal sources for our analyses [7]; however, they were unavailable. Thus, we selected skin fibroblast cells to perform proteomic analysis for two reasons. First, TGCV is a substantially rare disease and the only patient-derived cells found so far to act as proteomic resources are two fibroblasts. Second, patient-derived skin fibroblast cells have been utilized by several groups to study the features of NLSDM and showed intracellular deposition of lipid droplets [1,5]. Indeed, we confirmed that TGCV patient cells accumulated lipid droplets as described later (Figure 1B).

SILAC is a metabolic protein labeling method that employs isotopic variants of amino acids added to the growth medium [8]. Most commonly, different populations of cells are grown in medium containing distinct forms of both arginine (Arg) and lysine (Lys). SILAC analysis facilitates the relative quantification of small changes in protein abundance in cells and allows for the discovery of novel cellular pathways that are altered in disease progression.

In this study, we used the triple SILAC method in which two patient cells were labeled with distinct isotopic forms of amino acids. A schematic experimental design is shown in Figure 1A. In short, fibroblasts derived from two different patients, LC1 and LC2, were grown in medium (M) and heavy (H), respectively, and three control fibroblasts (control1, control2 and control3) were independently grown in light (L). Proteins extracted from the three control cells were mixed at the same protein concentration and used as a control for proteomic analysis. Then, proteins from the three labeled cell populations (LC1-M, LC2-H, and control -L) were mixed equally, followed by separation with SDS-PAGE. After separating the proteins, the gel was excised into 46 sections and proteins were in-gel digested with trypsin, extracted from gel pieces and analyzed by LC-MS/MS.

It was not immediately clear whether the dialyzed FBS, commonly used for SILAC labeling as described in the Methods section, would maintain the lipid deposition of patient cells. Thus, we examined whether the labeling procedure affected the lipid droplet deposition of patient cells in SILAC medium. After more than six doublings, which were sufficient for complete incorporation of the isotopic form of amino acids, patient cells remained in the lipid droplet deposition state, as demonstrated by Oil Red O staining (Figure 1B).

We were able to identify 4570 proteins with a false discovery rate (FDR) for protein identification of 1%, determined by searching a reverse database, and among the identified proteins, 4033 could be quantified. A list of all identified proteins is presented in Additional file 1: Table S1.

**Figure 2:**
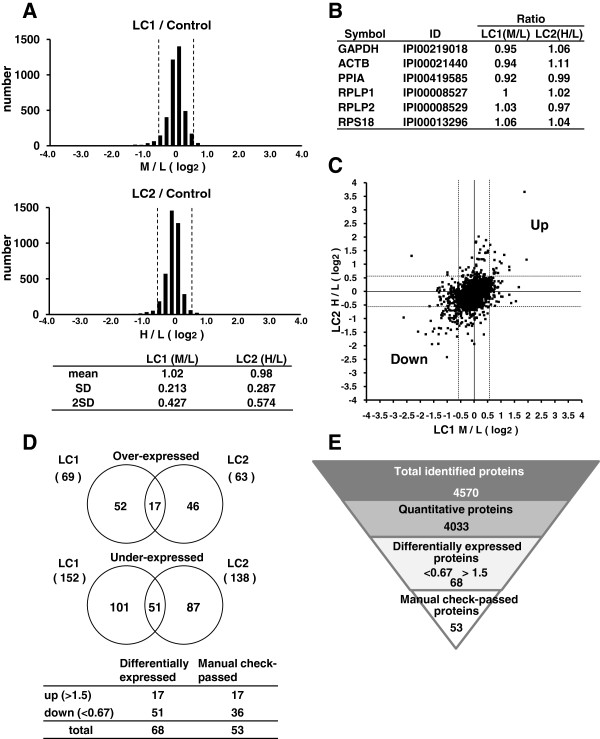
**Statistical analysis of SILAC data of two patient cells. (A)** Upper panels, log2 distributions of Medium (M)/Light (L) and Heavy (H)/Light (L) ratios of proteins quantified in SILAC analysis. Dashed lines represented log2 transformed cut-off values (>1.5, <0.67). Lower panel, statistical summary of ratios of two patient cells. **(B)** Ratios of various housekeeping proteins identified in SILAC analysis. **(C)** Scatter plot of log2 transformed patient cells/control cells ratios. X- and Y- axes represent log2 transformed M/L and H/L ratios, respectively, as indicated in the graph. **(D)** Upper panels, Venn diagrams show the number of overlapping altered proteins between the two patient cells. Lower panel, if there were only two quantified peptides, individual peptides must have met the cut-off value (>1.5 or <0.67) and proteins found not to meet this criteria were discarded. “Manual check-passed” represents remaining proteins after implementing the above criteria. **(E)** Summary of quantitative proteomics.

Prior to analyzing differentially expressed proteins, we plotted log2 transformed M/L (LC1/control) and H/L (LC2/control) ratios of all quantified proteins. Histograms of both LC1 and LC2 ratios showed normal Gaussian distribution centered around zero (Figure 2A). These normal distributions indicated that labeling processes by two distinct isotopic forms, M and H, did not produce a bias. In addition, various housekeeping proteins, including GAPDH, ACTB, PPIA, RPLP1, RPLP2 and RPS18, all exhibited ratios around 1 (Figure 2B).

To find differentially expressed proteins in patient cells, we determined the cut-off values for LC1 and LC2 data sets. The cut-off value of 1.5 for both LC1 and LC2 was selected, because 1.5 was approximately the mean plus two standard deviations (Figure 2A, lower panel). A logarithmic (log2) plot of the SILAC ratios H/L (X-axis) and M/L (Y-axis) for each quantified protein allows the unbiased identification of proteins that are specifically differentially expressed in patient cells (Figure 2C). Almost all proteins identified had ratios of around 1:1, and thus clustered around the intersection of the X- and Y-axes. Proteins over-expressed in both LC1 and LC2 have a high ratio of both M/L and H/L and could therefore be identified as outliers in the top right quadrant. In contrast, proteins under-expressed in both cells have a low ratio of both M/L and H/L and were identified as outliers in the bottom left quadrant.

Using cut-off values (>1.5 or <0.67), 69 and 152 proteins were found to be over- and under-expressed, respectively, in LC1 cells, and 63 and 138 proteins were found to be over- and under-expressed, respectively, in LC2 cells compared to control cells (Figure 2D, upper panel). To ensure that the observed changes of protein expression in patient cells were the result of TGCV, we considered stringent criteria. Each of the changed proteins had to meet cut-offs in both LC1 and LC2. Thus, we excluded any proteins found to meet the cut-off value in one patient cell data set but not in the other. After implementing the above stringent criteria, 17 and 51 proteins remained as over- and under-expressed, respectively, in both patient cells.

Furthermore, we manually checked the actual identified peptide ratios of each differentially expressed protein. If there were only two quantified peptides, individual peptides must have met the cut-off value (>1.5 or <0.67) and proteins found not to meet this criteria were discarded. Finally, 17 and 36 proteins remained as over- and under-expressed, respectively (Figure 2D, lower panel). A summary of SILAC proteomics is shown in Figure 2E. The quantification results for all the differentially expressed proteins measured in LC1 and LC2 cells are summarized in Table 1.

**Table 1:** Differentially expressed proteins in TGCV patient cells

**Protein description**	**Symbol**	**ID**	**Ratio**		
			**LC1 (M/L)**	**LC2 (H/L)**	**Ave**
**Over-expressed proteins**					
Filaggrin	FLG	IPI00026256	3.66	12.67	8.17
Collagen triple helix repeat containing 1	CTHRC1	IPI00060423	3.86	2.25	3.06
Aldehyde dehydrogenase 1 family, member B1	ALDH1B1	IPI00103467	1.51	3.28	2.4
Ribosomal protein S4, Y-linked 1	RPS4Y1	IPI00302740	2.17	2.54	2.36
Integrin, alpha 11	ITGA11	IPI00215613	1.71	2.73	2.22
Perilipin 2	PLIN2	IPI00293307	2.47	1.87	2.17
Molybdenum cofactor sulfurase	MOCOS	IPI00304895	2.48	1.72	2.1
DEAD (Asp-Glu-Ala-Asp) box polypeptide 3, Y-linked	DDX3Y	IPI00293616	1.94	2.14	2.04
LIM and cysteine-rich domains 1	LMCD1	IPI00303258	2.02	2.05	2.04
Integrin, alpha 6	ITGA6	IPI00010697	1.69	2.23	1.96
Nicotinamide N-methyltransferase	NNMT	IPI00027681	1.78	1.81	1.8
Glutamine-fructose-6-phosphate transaminase 2	GFPT2	IPI00216159	1.85	1.6	1.73
Membrane metallo-endopeptidase	MME	IPI00247063	1.64	1.77	1.71
Met proto-oncogene (hepatocyte growth factor receptor)	MET	IPI00294528	1.67	1.7	1.69
BAI1-associated protein 2	BAIAP2	IPI00299088	1.73	1.62	1.68
TSC22 domain family, member 2	TSC22D2	IPI00477806	1.68	1.57	1.63
von Willebrand factor A domain containing 8	VWA8	IPI00900366	1.66	1.59	1.63
**Under-expressed proteins**					
Mitochondrial ribosomal protein S28	MRPS28	IPI00795922	0.24	0.36	0.3
Collagen, type XVIII, alpha 1	COL18A1	IPI00783931	0.34	0.26	0.3
Armadillo repeat containing 9	ARMC9	IPI00829927	0.29	0.34	0.32
Fatty acid binding protein 3, muscle and heart (mammary-derived growth inhibitor)	FABP3	IPI00219684	0.4	0.26	0.33
Transient receptor potential cation channel, subfamily V, member 2	TRPV2	IPI00183666	0.16	0.51	0.34
Collagen, type VIII, alpha 1	COL8A1	IPI00942464	0.35	0.36	0.36
5'-nucleotidase domain containing 3	NT5DC3	IPI00465170	0.4	0.34	0.37
Gremlin 1, DAN family BMP antagonist	GREM1	IPI00298476	0.36	0.39	0.38
Dipeptidyl-peptidase 4	DPP4	IPI00018953	0.4	0.39	0.4
EH-domain containing 3	EHD3	IPI00021458	0.33	0.47	0.4
Cellular retinoic acid binding protein 2	CRABP2	IPI00216088	0.49	0.34	0.42
Aldehyde dehydrogenase 3 family, member A2	ALDH3A2	IPI00394758	0.55	0.38	0.47
Tubulin, alpha 4a	TUBA4A	IPI00007750	0.43	0.51	0.47
Prostaglandin-endoperoxide synthase 1 (prostaglandin G/H synthase and cyclooxygenase)	PTGS1	IPI00298267	0.65	0.32	0.49
Peroxiredoxin 6	PRDX6	IPI00220301	0.44	0.54	0.49
Argininosuccinate synthase 1	ASS1	IPI00020632	0.44	0.57	0.51
Nudix (nucleoside diphosphate linked moiety X)-type motif 2	NUDT2	IPI00221231	0.54	0.48	0.51
Fatty acid desaturase 2	FADS2	IPI00183786	0.47	0.56	0.52
Collagen, type VI, alpha 3	COL6A3	IPI00072917	0.65	0.38	0.52
Opioid growth factor receptor	OGFR	IPI00021537	0.53	0.5	0.52
Sulfide quinone reductase-like (yeast)	SQRDL	IPI00009634	0.58	0.46	0.52
Farnesyl-diphosphate farnesyltransferase 1	FDFT1	IPI00020944	0.45	0.61	0.53
Acyl-CoA oxidase 2, branched chain	ACOX2	IPI00293125	0.61	0.46	0.54
Phosphodiesterase 1C, calmodulin-dependent 70 kDa	PDE1C	IPI00028928	0.53	0.54	0.54
NME/NM23 nucleoside diphosphate kinase 3	NME3	IPI00012315	0.65	0.44	0.55
Laminin, beta 1	LAMB1	IPI00013976	0.59	0.5	0.55
Aldo-keto reductase family 1, member B1 (aldose reductase)	AKR1B1	IPI00413641	0.48	0.64	0.56
Chondroitin sulfate proteoglycan 4	CSPG4	IPI00019157	0.65	0.48	0.57
Glycoprotein (transmembrane) nmb	GPNMB	IPI00470529	0.6	0.53	0.57
Tweety homolog 3 (Drosophila)	TTYH3	IPI00749429	0.53	0.63	0.58
S100 calcium binding protein A4	S100A4	IPI00032313	0.54	0.62	0.58
Paraoxonase 2	PON2	IPI00290945	0.51	0.65	0.58
24-dehydrocholesterol reductase	DHCR24	IPI00016703	0.63	0.57	0.6
Potassium channel tetramerisation domain containing 12	KCTD12	IPI00060715	0.65	0.57	0.61
Mucosa associated lymphoid tissue lymphoma translocation gene 1	MALT1	IPI00009540	0.66	0.61	0.64
Phospholipase C, delta 3	PLCD3	IPI00152701	0.64	0.67	0.66

### Function and network analysis of identified proteins

**Figure 3:**
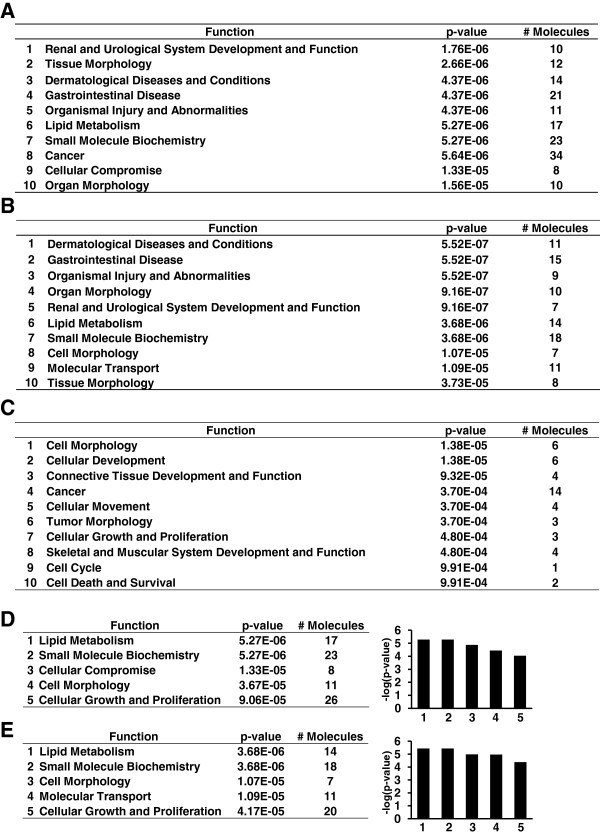
**Top bio-functions of identified proteins in SILAC proteomic analysis.** Functions are generated by IPA analysis and displayed according to statistical significance values. **(A-C)** Top 10 global functions identified by three data sets. **(A)** Functions of all differentially expressed proteins in TGCV patient cells. **(B)** Functions of under-expressed proteins in TGCV patient cells. **(C)** Functions of over-expressed proteins in TGCV patient cells. **(D)** Left panel, individual functions assigned in Molecular and Cellular Functions category by using all differentially expressed proteins in TGCV patient cells. Right panel, histogram according to p-values described in left panel. **(E)** Individual functions assigned in Molecular and Cellular Functions category using under-expressed proteins, the same format as described in **(D)**.

To reveal significant networks relevant to the pathogenesis of TGCV, we next imported the identified proteins list into IPA. First, we focused on “Lipid Metabolism” in the functional distribution of identified proteins. Figure 3 shows the bio-functions of three data sets. Using the data set for all altered proteins, IPA identified “Lipid Metabolism” with higher significance, which ranked as a top group (Figure 3A). This result indicated that lipid metabolism-related proteins were specifically regulated in TGCV patient cells and our proteome analysis was not biased under experimental conditions. When the under-expressed proteins set was used, IPA also identified similar functions to those of all altered proteins and “Lipid Metabolism” was also ranked as a top group (Figure 3B). Therefore, under-expressed proteins might represent a functional change in TGCV patient cells. In contrast, when the over-expressed proteins set was used, IPA indicated different functions and “Lipid Metabolism” was ranked lower (Figure 3C).

Furthermore, we focused on category-specific bio-functions and discovered that “Lipid Metabolism” was top-ranked in Molecular and Cellular Functions in both all altered proteins and the under-expressed proteins sets (Figure 3D and E). Thus, it appeared that “Lipid Metabolism” might be entirely influenced by a loss-of-function mutation of ATGL. With regard to individual functions in “Lipid Metabolism”, “lipid synthesis” was found to be the most significant (P = 5.76E-06) and repressed (activation Z score = −2.02) function, which consisted of ACOX2, AKR1B1, DHCR24, DPP4, FABP3, FADS2, FDFT1, ITGA6, PLIN2, PON2, PTGS1, TRPV2, most of which have been previously reported to be associated with metabolic disease, as discussed later (Table 2) [14-23]. It should be noted that the function “Concentration of triacylglycerol” was identified in the all altered proteins data set and activated (activation Z score = 1.45) (Table 2). This might have been mostly owing to the presence of PLIN2, a lipid droplet maintenance protein described later. These results indicated that our proteomics analysis could usefully identify proteins specific for lipid metabolism aberration.

**Table 2:** Individual functions and identified molecules in “Lipid Metabolism” in differentially expressed proteins in TGCV cells

**Bio-functions**	**Functions annotation**	**p-value**	**Activation z-score**	**Molecules**	**Number of molecules**
Lipid metabolism	Synthesis of lipid	5.27E-06	−2.021	ACOX2, AKR1B, DHCR24, DPP4, FABP3, FADS2, FDFT1, ITGA6, PLIN2, PON2, PTGS1, TRPV2	12
Lipid metabolism	Concentration of lipid	9.56E-05	0.117	AKR1B1, COL18A1, CSPG4, DHCR24, FABP3, FADS2, FDFT1, PLIN2, PON2, PRDX6, PTGS1	11
Lipid metabolism	Concentration of fatty acid	2.53E-04	−0.85	AKR1B1,FABP3,FADS2,PON2,PRDX6,PTGS1	6
Lipid metabolism	Quantity of lipid peroxide	8.88E-04		PON2,PRDX6	2
Lipid metabolism	Conversion of lipid	1.13E-03	−1.091	DHCR24,MET,PRDX6,PTGS1	4
Lipid metabolism	Concentration of arachidonic acid	1.59E-03		FADS2,PTGS1	2
Lipid metabolism	Transport of long chain fatty acid	2.49E-03		FABP3,PLIN2	2
Lipid metabolism	Concentration of triacylglycerol	2.62E-03	1.446	AKR1B1,COL18A1,FADS2,PLIN2,PON2	5
Lipid metabolism	Accumulation of phospholipid hydroperoxide	2.97E-03		PRDX6	1
Lipid metabolism	Degradation of colfosceril palmitate	2.97E-03		PRDX6	1

Next, we systematically evaluated the functions of identified proteins by investigating the results of network analysis. Five networks were found to be significant using the all altered proteins data set, of which two networks were highly significant, with scores greater than 30 (Additional file 2: Table S2A). The major functions of network1 were “Dermatological Diseases and Conditions”, “Cancer” and “Renal Urological System Development and Function”. Specifically, the top function, “Dermatological Diseases and Conditions”, was represented by burn which was the most significant bio-function (P = 4.31E-06) (Additional file 2: Table S2B). This bio-function consisted of COL18A1, COL6A3, COL8A1 and PTGS1, most of which have been previously described to be associated with connective tissue function and structure (Additional file 2: Table S2B) [24-26]. With regard to under-expressed proteins, the major functions of network1 were “Dermatological Diseases and Conditions”, “Gastrointestinal Disease” and “Organismal Injury and Abnormalities” (Additional file 2: Table S2C and D). Similar to the all altered proteins data set, assigned proteins were involved in connective tissue function.

On the other hand, using only the over-expressed proteins data set, IPA identified one network with a score greater than 30. Major functions of this network were “Cell Morphology”, “Cellular Development” and “Cancer”, which were not uncommon results from this sort of analysis. It seemed that this result is mostly influenced by only a few proteins, due to the relatively low number of over-expressed proteins. These results were in good agreement with the above-mentioned global functional distribution (Figure 3A, B and C).

### Confirmation of differentially expressed proteins using SRM/MRM analysis

To ensure that the difference in protein expression between TGCV patient cells and control cells results from TGCV-specific processes, we performed to confirm the differentially expressed proteins by SRM/MRM analysis using fibroblast cell lysates cultured in normal medium. For SRM/MRM analysis, the top 10 over- and under-expressed proteins were selected. Target peptides for measurement were chosen using the criteria below. Peptides were unique proteotypic peptide sequences (typically 7–20 amino acids) based on the results obtained from shotgun proteomics and did not possess methionine in order to avoid partial oxidation. When possible, two peptides were used per protein. Finally, we selected 42 peptides representing 20 differentially expressed proteins. Complete information (so-called transition list) about the selected peptides is presented in Additional file 3: Table S3.

**Figure 4:**
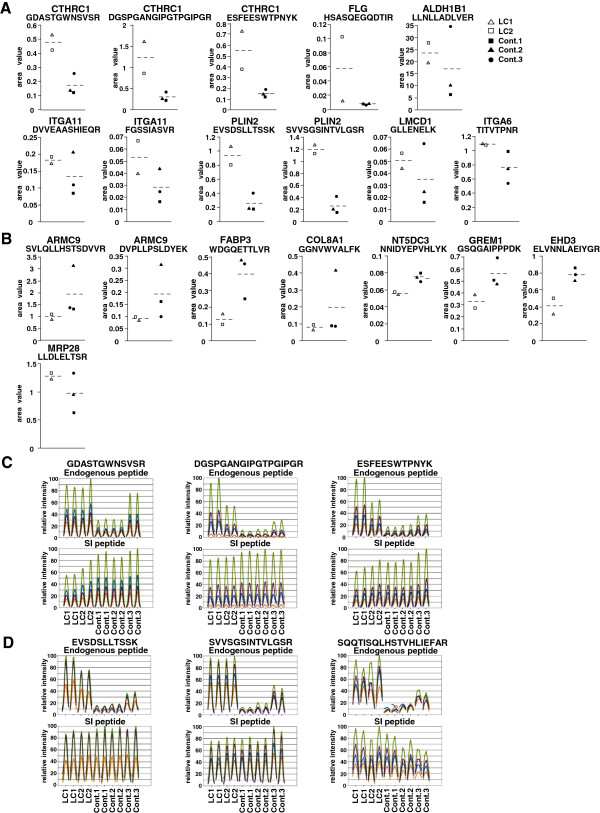
**SRM analysis confirms differential expression of proteins between TGCV patient and control cells. (A)** Seven of top 10 over-expressed proteins were successfully quantified in SRM analysis, showing the same trend as in SILAC proteomic analysis. **(B)** Seven of top 10 under-expressed proteins were successfully quantified. These proteins showed the same trend as in SILAC proteomic analysis, except for one protein. Y-axis indicates the peak area ratio of the endogenous peptide to SI-peptide. Protein names and target peptide sequences for SRM are indicated at the top of each graph. Symbols represent each cell used in SRM analysis, as described in the figure. **(C** and **D)** Examples of peak groups of each peptide transition obtained in SRM analysis. The peak areas from each chromatogram are displayed in parallel and represent detected ion intensities. Colors indicate the different transitions of each peptide. **(C)** CTHRC1: Three endogenous target peptides were quantified. **(D)** PLIN2: Two endogenous target peptides were quantified (left and middle panels). One peptide was not quantified due to a low signal-to-noise ratio in control1 (right panel). Target peptide sequences are indicated at the top. Characters at the bottom indicate cell names. Analyses were run in duplicate.

Whole cell lysates of fibroblast cells were digested by trypsin and monitored by SRM/MRM analysis in duplicate. Consequently, SRM/MRM analysis successfully quantified 7 of over-expressed and 7 of under-expressed proteins (Figure 4). Among them, 13 proteins showed a trend in the same direction, despite a modest change under different cell conditions (Figure 4A and B). In addition, we focused on 2 proteins, PLIN2 and CTHRC1, because these proteins were possible biomarker candidates in the context of lipid metabolism. Therefore, one more peptide was selected to confirm the difference. CTHRC1, which has been reported to be involved in lipid metabolism [27], successfully showed apparent changes in all three target peptides (Figure 4A and C). PLIN2, which has been reported to be involved in the maintenance of lipid droplets [22,28], also showed apparent changes in all three target peptides (Figure 4A and D).

### Quantitative transcriptomic analysis of TGCV patient cells

To address the relationship of protein expression levels with transcript levels, we performed oligonucleotide microarray analysis using Affymetrix microarrays of Human Genome U133 Plus 2.0. A total of 26780 mRNA species were quantified, of which 20743 were deemed “present” in all three RNA samples. A list of 20743 transcripts is presented in Additional file 4: Table S4. Histograms of both LC1 and LC2 log2 ratios showed normal distribution centered at zero, similar to proteome analysis (Additional file 5: Figure S1A). These normal distributions also indicated that most transcript levels were unaffected by labeling processes by two distinct isotopic forms of amino acids. In addition, various housekeeping genes, including *GAPDH*, *ACTB*, *TBP*, *PPIA*, *RPS18*, *B2M*, *RPLP1* and *RPLP2*, all exhibited ratios around 1 (Additional file 6: Table S5).

A logarithmic (log2) plot of ratios LC1/control (X-axis) and LC2/control (Y-axis) for each quantified gene indicates the unbiased identification of genes that are specifically differentially expressed in patient cells (Additional file 5: Figure S1B). Although almost all genes identified had ratios of around 1:1, the scatter plot at transcription levels showed dispersion of the points around the regression line (R^2^ = 0.323) compared with that of protein levels. Transcriptomic analysis tends to show differences between patient and control cells due to a greater number of identified transcripts than proteins in proteomic analysis. We considered a ratio of 2.0 as the cut-off for differentially expressed genes and applied stringent criteria similarly to proteome analysis; the changed transcripts must have met cut-offs in both LC1 and LC2. Among 20743 transcripts, 130 and 122 genes remained as over- and under-expressed, respectively, in both patient cells (Additional file 7: Table S6).

### Confirmation of differentially expressed genes using real-time RT-PCR (quantitative PCR) analysis

To further confirm the gene expression results from array-based measurements, we performed quantitative PCR (qPCR) using RNA isolated from cells cultured in normal medium. We selected the top 5 over- and under-expressed genes for confirmation. Quantitative PCR revealed that 9 out of 10 showed the same direction as array-based measurements (Additional file 8: Figure S2). All five genes, highly expressed in array analysis, showed apparently increasing RNA expression in qPCR (Additional file 8: Figure S2A). In particular, *FBN2*, *FGL2* and *FLG* showed RNA expression levels well consistent with array-based results. On the other hand, 4 out of 5 under-expressed genes showed apparently decreasing RNA expression in qPCR (Additional file 8: Figure S2B). These results indicated that array analysis might achieve sufficient analytical completeness.

### Comparison of protein expression with gene expression levels

**Figure 5:**
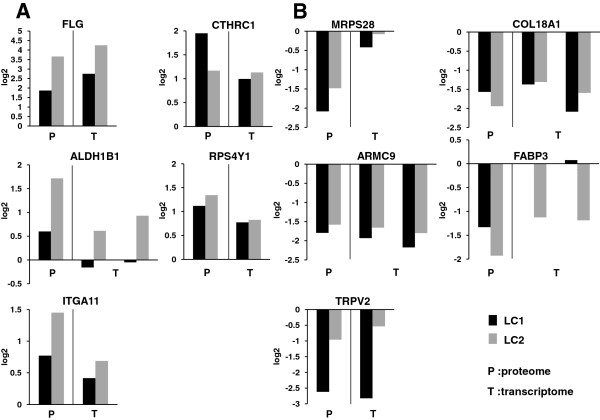
**Protein and gene expression levels for the top 5 over- and under-expressed proteins in SILAC analysis. (A** and **B)** Comparison of expression levels of top 5 over- **(A)** and under- **(B)** expressed proteins in SILAC analysis with those of microarray transcriptomic analysis. Y-axis represents log2 transformed ratio in both proteome and transcriptome. Black bars and gray bars indicate LC1 and LC2 cells, respectively, as indicated in the figure. Pair of bars on left and right of each graph represent log2 ratios of proteome and transcriptome, respectively, as indicated at the bottom. When a transcript was detected with more than one probe set, each ratio was represented by separate pairs of bars.

For the top 5 over- and under-expressed proteins in SILAC analysis, the proteomic and transcriptomic data are shown (Figure 5A and B). Among them, 8 proteins had consistent expression profiles between protein and mRNA levels, even though no change of *ALDH1B1* and *FABP3* was observed in only LC1 at mRNA levels. Thus, the results of our proteomic analysis were further supported by RNA expression levels, at least for these proteins.

**Figure 6:**
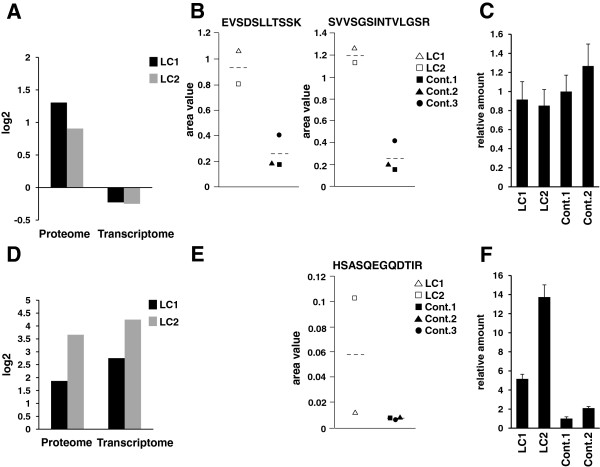
**SRM and qPCR analysis of two selected differentially expressed proteins. (A)** Comparison of PLIN2 expression in SILAC proteomics with that of microarray transcriptomics using the same format as described in the legend of Figure 5. PLIN2 expression is elevated at protein levels but not transcript levels. **(B)** SRM analysis confirmed over-expression of PLIN2 at protein levels. **(C)** qPCR analysis confirmed that expressions of *PLIN2* were not so different at transcript levels. Characters at the bottom indicate cell names. **(D)** Comparison of FLG expression in SILAC analysis with that of microarray transcriptomics using the same format as described in the legend of Figure 5. **(E)** SRM analysis confirmed over-expression of FLG at protein levels. **(F)** Quantitative PCR analysis confirmed over-expression of *FLG* at transcript levels. Characters at the bottom indicate cell names.

First, we focused on PLIN2 expression, which is a well-known protein that coats lipid droplets in multiple non-adipose tissues, as mentioned above [22]. The proteomic and transcriptomic data for PLIN2 are shown in Figure 6A. The gene products showed no change or less than that of control cells at the transcriptome level (Figure 6A). These data might be of interest for further investigation of the role of PLIN2 in TGCV pathogenesis. Validation using qPCR verified relatively similar expression levels between patient cells and control cells (Figure 6C). These results suggested that the stability of PLIN2 might increase at the protein level when lipid droplets were developing and revealed that posttranslational controls are important regulators of PLIN2 functions.

Next, we examined filaggrin expression, the most elevated molecule at the protein level, which is well-known to contribute to skin barrier formation [29]. The proteomic and transcriptomic data for filaggrin are shown in Figure 6D. Quantitative PCR analysis confirmed the elevated expression of *FLG* mRNA in good agreement with the proteomic and transcriptomic data (Figure 6F). SRM/MRM also confirmed the same trend with the proteomic data (Figure 6E). Filaggrin is of interest in the context of the pathogenesis of TGCV and is discussed in a later section.

### Function and network analysis of identified genes

We imported the differentially expressed genes list into an IPA search. Five networks were identified using the all altered genes data set, of which 4 networks were highly significant with scores greater than 30 (Additional file 9: Table S7A). The major functions of network1 were “Connective Tissue Disorder”, “Developmental Disorder” and “Liver Cirrhosis”. The top function included in network1 was represented by *COL18A1*, *CSPG4*, *FBLN1*, *GATA6, LAMA1*, *LAMA2*, *MMP1*, *OLR1*, *PLA2G4a* and *TGFB2*, most of which have been previously reported to be closely associated with connective tissue function and structure (Additional file 9: Table S7B) [26,30-36]. These results were in good agreement with the changes of connective tissue functions observed in proteomics analysis.

## Discussion

Genetic mutations in ATGL are known causes of TGCV, but the pathogenesis mechanism remains unclear. Here we examined proteomic profiles of skin fibroblast cells derived from patients to elucidate pathogenesis processes and find clinically relevant proteins for TGCV. We identified over 4500 proteins, and specified over 50 proteins to be differentially expressed between TGCV and healthy control cells. To our knowledge, this is the most comprehensive quantitative study to date, aiming to understand the pathogenesis mechanisms of ATGL deficiency. We have provided a comprehensive proteome database that is essential to discover the key molecules underlying ATGL deficiency.

The main findings of our study are as follows. (1) Bio-function analysis revealed that proteins associated with “Lipid Metabolism” were affected by the cellular status of TGCV. Those proteins, such as DPP4, PON2, PTGS1 and PLIN2, are known to be key molecules in the metabolic process. (2) Network analysis revealed that the main network of altered proteins and genes in TGCV cells was tightly associated with connective tissue disorders. (3) Candidate proteins responsible for TGCV pathogenesis were discovered, such as PLIN2, CTHRC1 and FLG, and their altered expressions were confirmed by SRM/MRM and qPCR.

IPA analysis identified “Lipid Metabolism” as the top-ranked bio-function of altered proteins in TGCV cells. Proteins involved in this function are well-known key molecules in the metabolic process, as described in the Results section (Table 2) and are therefore candidate targets of therapy. For example, DPP4 is a serine protease, that specifically degrades incretin hormones; thus, its inhibitor is considered as a useful drug for type 2 diabetes mellitus [37]. Recently, DPP4 inhibition was also reported to be associated with an improved cardiovascular profile, although its precise role is still unknown [38]. PON2 possesses anti-oxidant properties, protecting cells from oxidative stress [39] and has been suggested to exert protection against macrophage TG accumulation and oxidative stress [40]. PTGS1, also known as cyclooxygenase 1, plays a pivotal role in the biosynthesis of prostanoids and thus regulation of PTGS1 activity has been considered in the therapy for various diseases [23,41]. Therefore, these proteins are considerable candidates as therapy targets for TGCV, even though their involvement in TGCV pathogenesis is unknown.

Reduced expression of PTGS1 was identified in both proteomic and transcriptomic analysis (Table 1 and Additional file 7: Table S6). In addition, reduced expression of PTGS2, which is the key enzyme in prostaglandin biosynthesis similar to PTGS1 [42], was also detected at the transcription level (Additional file 7: Table S6). In contrast, over-expression of PLA2G4A, which is the enzyme that releases arachidonic acid from the phospholipid [43], was observed at the transcription level and confirmed by qPCR (Additional file 7: Table S6 and Additional file 8: Figure S2). From these results, we speculate that aberration of the arachidonic acid cascade may occur in TGCV cells. However, further investigation is needed for elucidation.

IPA network analysis also revealed that the top-ranked networks of differentially expressed proteins and genes were tightly associated with connective tissue-related molecules (Additional file 2: Table S2 and Additional file 9: Table S7). For example, COL18A1, COL6A3, COL8A1, FBLN1, LAMA1, LAMA2 and CSPG4 are well-known extracellular matrix proteins [24-26,30,31,34]. TGCV patients showed marked characteristics, such as massive accumulation of TG in the skeletal, heart muscles and coronary artery. Aberration of molecules composed of connective tissues appeared to be involved in this symptom, but its precise role in the pathogenesis is still unclear and awaits further investigation.

Of the top-ranked over-expressed proteins, we focused on three proteins, PLIN2, CTHRC1 and FLG, whose over-expression was successfully confirmed by SRM/MRM. These proteins are interesting in the context of lipid metabolism disorders as discussed below.

PLIN2, is well-known to play a role in the formation of intracellular lipid droplets, and therefore to promote neutral lipid stores, particularly in non-adipose tissues [22]. It exhibited a 2.1-fold increase in TGCV patient cells and was successfully confirmed in SRM/MRM analysis (Table 1, Figure 4A and D). Straub et al. described that PLIN2 was a general marker for a variety of human diseases associated with lipid droplet accumulation [28]. In this study, we showed that PLIN2 was over-expressed in TGCV patient cells at the protein level, which was in good agreement with previous studies in the context of lipid storage diseases. In contrast, results from both array and qPCR analysis indicated that PLIN2 expression did not differ at mRNA levels between TGCV patient and control cells (Figure 6). From these results, we suggest that PLIN2 may be stabilized at the protein level during the progression of lipid droplets in TGCV patient cells. A possible reason is that PLIN2 was protected from the proteolysis pathway through the interaction with lipid droplets. However, more studies are needed to reach a firm conclusion.

One of the top-ranked over-expressed proteins, CTHRC1, which was 3-fold elevated in patient cells, was successfully confirmed in SRM/MRM analysis (Figure 4A and C). CTHRC1 is a secreted protein that has activity to repress collagen matrix synthesis during vascular remodeling [44]. Therefore, it appears to be involved in the generation of atherosclerosis lesions in TGCV. Interestingly, a recent study by Stohn et al. identified CTHRC1 as a novel circulating protein having hormone-like metabolic effects [27]. Livers from CTHRC1 null mice accumulated vast quantities of lipid, leading to extensive macrovesicular steatosis. Thus, CTHRC1 is likely to have the ability to repress the generation of lipid droplets and be over-expressed as it reflects the deposition of TG in TGCV patient cells. It should be noted that CTHRC1 may be a possible candidate biomarker for TGCV as it is a secreted protein. However, its precise role in TGCV pathogenesis and value as a biomarker for TGCV await further investigation of patients.

The other top-ranked over-expressed protein, FLG, was 8-fold elevated (Table 1). FLG is a key molecule that facilitates terminal differentiation of the epidermis and formation of the protective skin barrier [45]. Lipid organization is well-known to play an important role in epidermal differentiation [46,47]. Thus, one question is whether FLG over-expression occurs as a result of a disorder of epidermal differentiation, which is affected by lipid metabolism failure. However, this might be contradictory. FLG acts as a major component in the outer layer of the epidermis [29,45]. On the other hand, skin fibroblast cells exist in the dermis, separated from the epidermis by a basement membrane. Therefore, it is unlikely that FLG over-expression in skin fibroblast cells is a result of a disorder of epidermal differentiation. Over-expression of FLG may rather be involved in TGCV pathological conditions, although the contributions of FLG to the TGCV pathogenesis process remain to be elucidated.

## Conclusions

Although numerous studies have been performed to elucidate the pathogenesis of rare diseases, few have succeeded, mainly due to the lack of comprehensive studies, which are difficult because the research is often limited by ethical issues involved in the use of patient materials. To overcome this difficulty and understand TGCV, we performed an SILAC- and SRM/MRM-based identification-through-confirmation study using skin fibroblast cells derived from two patients, which contributed to finding clinically worthwhile proteins for TGCV. This strategy provides rapid and comprehensive identification of differentially expressed proteins in TGCV patient cells.

Consequently, we were able to identify and quantify 4033 proteins, 53 of which showed significantly altered expression in TGCV patient cells. A complete quantified proteins list is available as Additional file 1: Table S1. In addition, a complete quantified transcripts list is available as Additional file 4: Table S4. Our findings will be useful to elucidate the pathogenic mechanism of TGCV and discover clinically relevant molecules for TGCV in the near future.

## Abbreviations

PBS: Phosphate-buffered saline; DTT: Dithiothreitol; LC-MS/MS: Liquid chromatography-tandem mass spectrometry; SI-peptide: Stable isotope labeled peptide; PCR: Polymerase chain reaction; GAPDH: Glyceraldehyde-3-phosphate dehydrogenase; ACTB: Actin, beta; PPIA: Peptidylprolyl isomerase A; RPLP1: Ribosomal protein, large, P1; RPLP2: Ribosomal protein, large, P2; RPS18: Ribosomal protein S18; ACOX2: Acyl-CoA oxidase 2, branched chain; AKR1B1: Aldo-keto reductase family 1, member B1; DHCR24: 24-dehydrocholesterol reductase; DPP4: Dipeptidyl-peptidase 4; FABP3: Fatty acid binding protein 3; FADS2: Fatty acid desaturase 2; FDFT1: Farnesyl-diphosphate farnesyltransferase 1; ITGA6: Integrin, alpha 6; PLIN2: Perilipin 2; PON2: Paraoxonase 2; PTGS1: Prostaglandin-endoperoxide synthase 1; TRPV2: Transient receptor potential cation channel, subfamily V, member 2; COL18A1: Collagen, type XVIII, alpha 1; COL6A3: Collagen, type VI, alpha 3; COL8A1: Collagen, type VIII, alpha 1; CTHRC1: Collagen triple helix repeat containing 1; TBP: TATA box binding protein; B2M: Beta-2-microglobulin; FBN2: Fibrillin 2; FGL2: Fibrinogen-like 2; FLG: Filaggrin; ALDH1B1: Aldehyde dehydrogenase 1 family, member B1; CSPG4: Chondroitin sulfate proteoglycan 4; FBLN1: Fibulin 1; GATA6: GATA binding protein 6; LAMA1: Laminin, alpha 1; LAMA2: Laminin, alpha 2; MMP1: Matrix metallopeptidase 1; OLR1: Oxidized low density lipoprotein (lectin-like) receptor 1; PLA2G4A: Phospholipase A2, group IVA; TGFB2: Transforming growth factor, beta 2; PTGS2: Prostaglandin-endoperoxide synthase 2.

## Competing interests

The authors declare no conflicts of interest.

## Authors’ contributions

YH performed experiments and analysis, and drafted the manuscript. NK performed experiments. YH and SW operated the SRM instrument. JA participated in the design of the study. KH participated in the design of the study and coordination, and helped to draft the manuscript. TT designed the study and wrote the manuscript. All authors read and approved the final manuscript.

## Supplementary Material

Additional file 1: Table S1 A list of identified and quantified proteins in SILAC proteomics analysis by MaxQuant software. Click here for file

Additional file 2: Table S2 Networks of differentially expressed proteins in TGCV cells. Click here for file

Additional file 3: Table S3 Sequences and SRM transitions of target peptide for 20 proteins. Click here for file

Additional file 4: Table S4 A list of identified and quantified transcripts by Human Genome U133 Plus 2.0 array. Click here for file

Additional file 5: Figure S1 Statistical analysis of microarray data of two patient cells. Click here for file

Additional file 6: Table S5 Ratios of various housekeeping genes observed in microarray analysis. Click here for file

Additional file 7: Table S6 Differentially expressed genes in TGCV patient cells. Click here for file

Additional file 8: Figure S2 Quantitative PCR confirms differential expression of genes between TGCV patient cells and control cells. Click here for file

Additional file 9: Table S7 Networks of differentially expressed genes in TGCV cells. Click here for file
