# Supplementary figures and images for: Quantitative proteomic analysis of cultured skin fibroblast cells derived from patients with triglyceride deposit cardiomyovasculopathy
Source: Orphanet J Rare Dis. 2013 Dec 21;8:197. doi: 10.1186/1750-1172-8-197 (PMC3891998; doi:10.1186/1750-1172-8-197)

**A**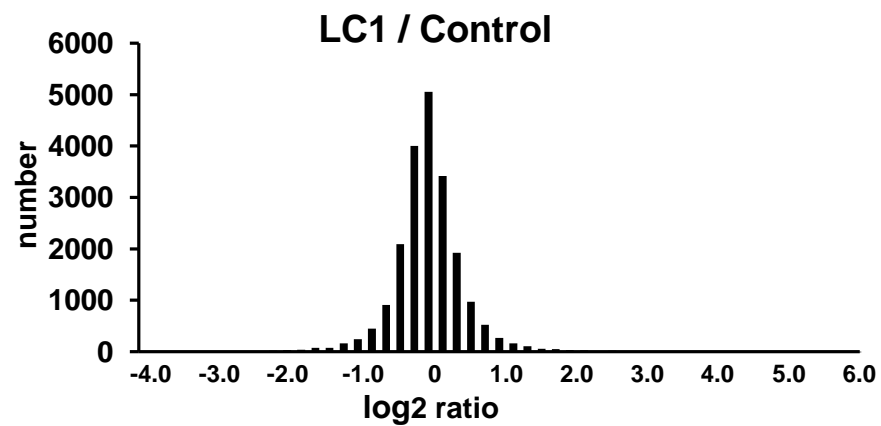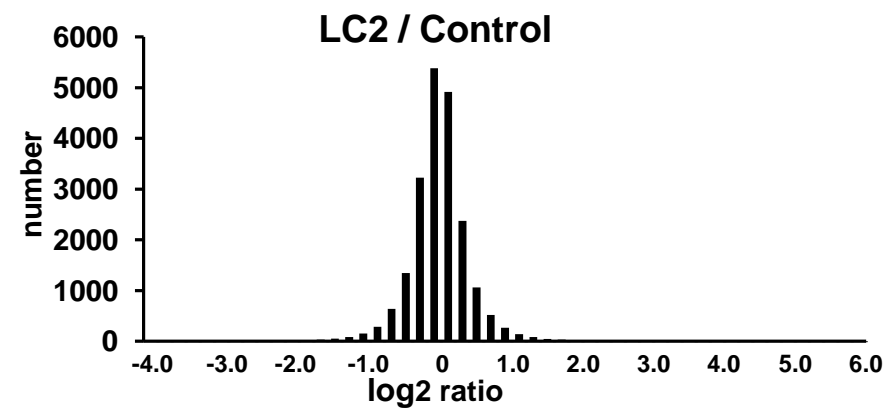**B**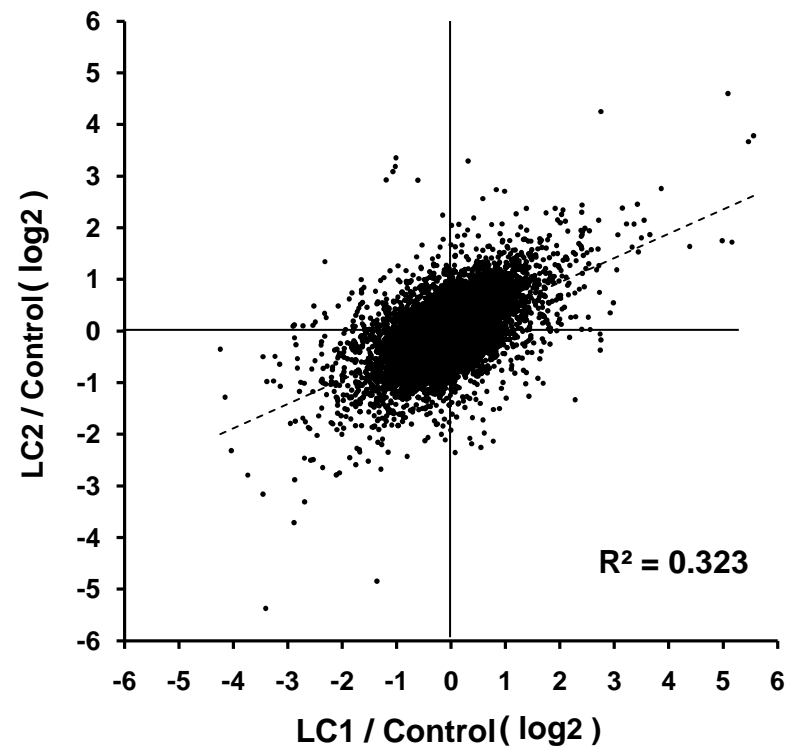

Supplement: Additional file 5: Figure S1 — Statistical analysis of microarray data of two patient cells. [file 1750-1172-8-197-S5.pdf]

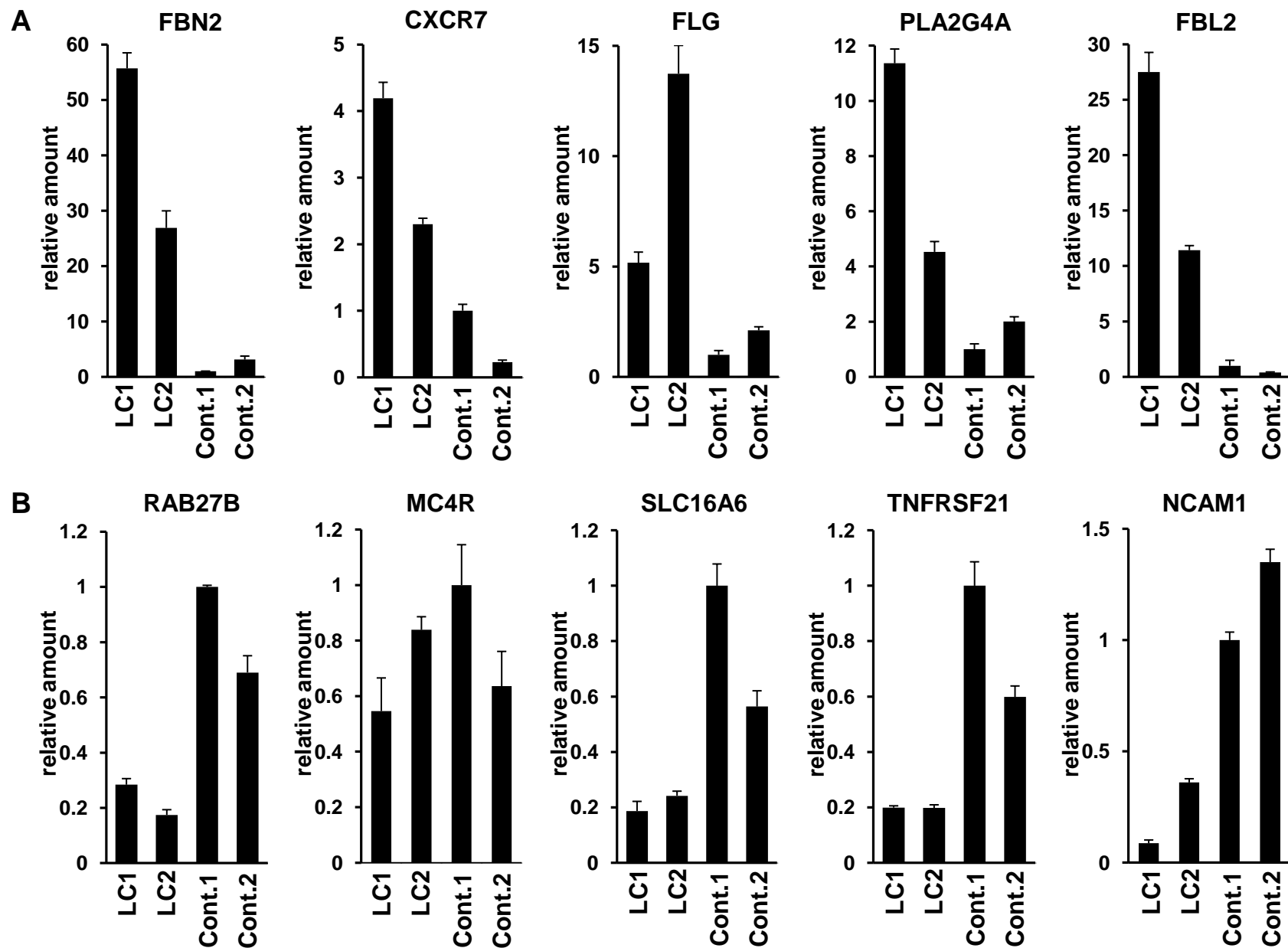

Supplement: Additional file 8: Figure S2 — Quantitative PCR confirms differential expression of genes between TGCV patient cells and control cells. [file 1750-1172-8-197-S8.pdf]
